# MicroRNA Expression Signatures Determine Prognosis and Survival in Glioblastoma Multiforme—a Systematic Overview

**DOI:** 10.1007/s12035-014-8668-y

**Published:** 2014-03-12

**Authors:** Michael Henriksen, Kasper Bendix Johnsen, Hjalte Holm Andersen, Linda Pilgaard, Meg Duroux

**Affiliations:** Laboratory for Cancer Biology, Institute of Health Science and Technology, Aalborg University, Fredrik Bajers Vej 3B, 9220 Aalborg Ø, Denmark

**Keywords:** MicroRNA, Glioblastoma multiforme, Glioma, Survival, Signature, Prognosis

## Abstract

Despite advances in our knowledge about glioblastoma multiforme (GBM) pathology, clinical challenges still lie ahead with respect to treatment in GBM due to high prevalence, poor prognosis, and frequent tumor relapse. The implication of microRNAs (miRNAs) in GBM is a rapidly expanding field of research with the aim to develop more targeted molecular therapies. This review aims to present a comprehensive overview of all the available literature, evaluating miRNA signatures as a function of prognosis and survival in GBM. The results are presented with a focus on studies derived from clinical data in databases and independent tissue cohorts where smaller samples sizes were investigated. Here, miRNA associated to longer survival (protective) and miRNA with shorter survival (risk-associated) have been identified and their signatures based on different prognostic attributes are described. Finally, miRNAs associated with disease progression or survival in several studies are identified and functionally described. These miRNAs may be valuable for future determination of patient prognosis and could possibly serve as targets for miRNA-based therapies, which hold a great potential in the treatment of this severe malignant disease.

## Introduction

Glioblastoma multiforme (GBM) is a severe type of brain cancer characterized by its large growth potential and very poor clinical outcome. It is one of the most aggressive and incurable types of cancer reflected in a median survival of less than 1 year of all GBM cases and a 5-year survival rate of less than 5 % [1, 2]. GBM affects 2–3 per 100,000 persons per year making it a rare type of cancer, but still, it accounts for 16 % of all brain tumors and 54 % of all clinically diagnosed gliomas in the USA [2]. The clinical presentation of GBM depends on the location of the tumors and generally involves focal neurological deficits, headaches, and seizures. Tumors are most commonly found in the frontal lobes of the supratentorial compartments; however, they are not restricted to these areas, as illustrated by GBM tumors found in other parts of the central nervous system (CNS), such as the spinal cord and brainstem [3].

GBM can be subdivided into de novo-occurring tumors, termed primary GBM, or tumors developed from lower-grade astrocytomas, termed secondary GBM. The most prominent occurring subtype of GBM is the primary tumors. These tumors are often characterized by amplification or overexpression of the epidermal growth factor receptor (EGFR) (40–60 % of all primary GBM tumors) combined with genetic alterations in the EGFR gene, which results in mutated forms of this receptor [1]. This is opposed to secondary GBM, which is characterized by progressive addition of mutations in p53, platelet-derived growth factor receptor, and the retinoblastoma gene [4–6]. Nevertheless, this distinctive division of mutations into the different GBM subtypes is not definitive [3].

Determination of disease prognosis is most often based on histological classification combined with information on patient age and tumor size and location. These factors have all been defined as indicators of patient survival and treatment outcome, but due to the sustained poor overall survival of GBM patients, new arrays of prognostic indicators have been requested to aid in the clinical decision making [1]. In recent years, several molecular biomarkers have been characterized including chromosomal aberrations, methylation status of the methyl guanine methyl transferase (MGMT) promoter, mutations in important genes (isocitrate dehydrogenase 1 (IDH1), EGFR, and p53), and dysregulation of microRNAs [7]. Loss of heterozygosity in chromosomes 9p and 10q is associated with decreased survival, while co-deletion of 1p and 19q correlates with better treatment response and longer survival [7]. Hypermethylation of the MGMT promoter leads to lower expression levels of MGMT, which sensitizes GBM tumors to chemotherapeutic treatment and thus is associated with a significantly better patient outcome [8, 9]. Improvement of the disease condition is also observed in patients with mutation in the IDH1 gene, which is most often found in secondary GBM. Furthermore, the expressional profile of specific microRNA signatures also correlates with overall survival, time to progression, and treatment success [10–12].

### The Fundamentals of MicroRNAs

MicroRNA (miRNA) is a class of non-coding single-stranded RNA comprised of approximately 22 nucleotides with the ability to negatively regulate gene expression posttranscriptionally [13, 14]. miRNAs bind to the 3′ untranslated regions (UTRs), and sometimes 5′UTRs, of their messenger RNA (mRNA) targets, to which they exhibit imperfect complementarity, hence, enabling one miRNA to inhibit translation of multiple genes [15, 16]. The first miRNA was discovered in 1993 in *Caenorhabditis elegans* and denoted lin-4 [17]. Later, upon the discovery of let-7, found to be conserved in several species, miRNA regulation was recognized as an omnipresent phenomenon in eukaryotic organisms [18, 19]. miRNAs are acknowledged as crucial micro-modulators of normal cellular homeostasis, and accordingly, dysregulation of miRNAs have been associated with a wide range of pathological conditions, such as cancer [20], cardiovascular disease [21, 22], and autoimmune [23] and neurodegenerative disorders [24]. Expression of miRNAs in pathological specimens or biofluids, compared to non-pathologic samples, is subject to great scientific efforts [25]. This poses interesting perspectives in terms of novel diagnostic and prognostic approaches and is inherently the initial step in uncovering the role of individual miRNAs in the context of different diseases, eventually paving the way for novel miRNA-based therapies.

### MicroRNA Biogenesis

**Fig. 1:**
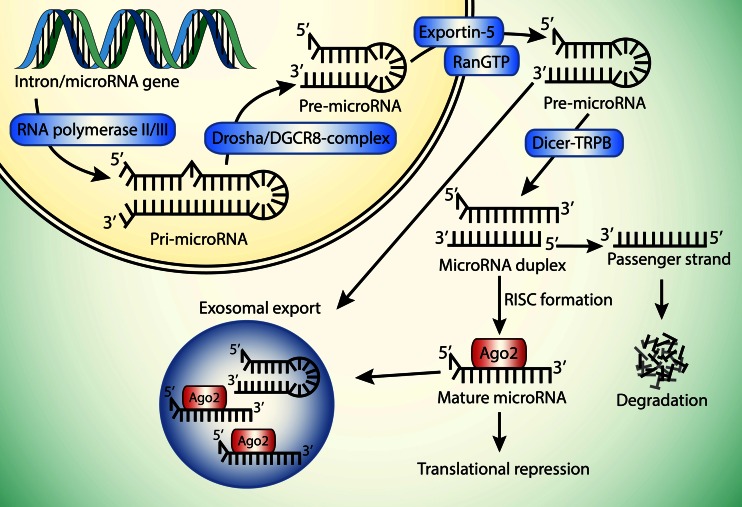
The biogenesis of miRNA requires RNA polymerase II/III for the transcription of pri-miRNA. The pri-miRNA product is then cleaved by the Drosha-DGCR8 complex into pre-miRNA. The pre-miRNA is exported to the cytoplasm by Exportin-5 in the presence of Ran-GTP co-factor. Once in the cytoplasm, the pre-miRNA is cleaved by the Dicer-TRBP complex into a miRNA duplex, which is unwound into two products: a guide strand bound to Ago2, which is incorporated into the RISC, and a passenger strand, which is degraded. Finally, the miRNA binds to its target mRNAs resulting in mRNA target cleavage, translational repression, or mRNA decay. A more novel fate of the miRNAs is the selective secretion via microvesicles or exosomes. Ran = Ras-related nuclear protein; GTP = guanosine-5′-triphosphate; TRBP = TAR (HIV-1) RNA binding protein; Ago2 = Argonaute protein 2; RISC = RNA-induced silencing complex

To understand the context of miRNA as a potential prognostic tool in patients with GBM, the essential steps in the biogenesis of miRNAs and the modes by which they exert their repression on downstream targets are summarized (see Fig. 1).

The linear biogenesis of miRNA begins with the transcription of miRNA genes by RNA polymerase II/III, giving rise to a primary transcript called pri-miRNA, which is subsequently polyadenylated and capped. The transcript then folds into a hairpin-loop structure via intrastrand base-pairing [26]. This structure is cleaved by the Drosha/DGCR8 complex to become pre-miRNA and transported out of the nucleus by Exportin-5 in a Ran-GTP-dependent process [27]. In the cell cytoplasm, the RNAse-III enzyme known as Dicer cleaves the pre-miRNA of which only one strand (known as guide strand) is incorporated into the RNA-induced silencing complex (RISC), the cytoplasmic effector machine of miRNA. The passenger strand is subsequently degraded [28]. The RISC is comprised of Dicer, double-stranded RNA-binding factor, and Argonaut protein 2 (Ago2). The posttranscriptional RNA silencing is facilitated via imperfect complementary binding of miRNA attached to RISC, to the respective mRNA 3′UTR, resulting in translational inhibition [29]. Additionally, miRNAs are selectively excreted via lipoproteins or microvesicles, potentially functioning as a mode of intercellular communication. This last notion is important in relation to the nature of sampling material in the sense that plasma miRNA patterns might be a useful diagnostic and/or prognostic marker of ongoing pathological processes [30, 31]. For a more comprehensive review of miRNA biogenesis, please refer to Winter et al. [26].

### MicroRNA Expression in Glioblastoma Multiforme

miRNAs can be regarded as cancer biomarkers when their variation in expression identifies the cancerous state. To date, almost all tumor tissue analyzed by miRNA profiling has provided distinct miRNA profiles compared to normal tissue [32]. These differential profiles can be further associated with prognostic factors and disease progression [33–35]. In GBM, the number of studies pertaining to miRNA expression and functional characterization has grown and miRNA signatures are refining GBM classification, differentiating between the different grades and stages, providing key regulatory links to disrupted signaling pathways such as those facilitating cell growth. This has lead to a more in depth understanding about GBM pathology [36].

Early studies show that miRNA expression in tumor samples seems lower, and this could be a function of cellular differentiation status [32, 37]. It appears that the most common dysregulation of miRNA in GBM is observed to be overexpression, based on the systematic literature review published by Møller et al. Here, for example, miR-17, miR-21, miR-93, miR-221, and miR-222 have been intensively investigated with respect to both their expression and functionality, but the functional properties of the vast majority remains completely unknown [38]. The most extensively investigated miRNA is miR-21, which is consistently reported to be overexpressed in GBM in a grade-specific manner [12, 39–68]. At least for GBM, miR-21 appears to be the major anti-apoptotic and pro-survival factor that is linked to shorter progression-free survival [12, 69, 70].

Expression profiling of miRNA in patient tissue and investigation of their putative function using in vitro primary cultures and in vivo studies have provided an insight not only into the genes that are regulated by respective miRNA, but also the pathways that are disrupted, many of which are hallmarks of GBM biology (reviewed by Lakomy et al. [12]). The pattern of miRNA expression, whether its up or downregulation, is now becoming a recognized tool in addition to gene expression profiling to stratify GBM patients into different groups [36]. Here, the miRNA cohorts are smaller and miRNA signatures pertaining to overall or progression-free survival are starting to evolve, albeit they are still very much dependent on the individual patient history, tumor size, age, and treatment regimen.

### Overall and Progression-Free Survival as Clinical Endpoints in Glioblastoma Multiforme

In the literature, both overall survival (OS) and progression-free survival (PFS) are widely used end points to assess the predictive factor of a given miRNA signature; however, the two terms do not provide equal information [71–73]. When evaluating a treatment response, OS is used as a measure of the end result including the complete disease history and possible other factors affecting the lifespan. The PFS is more specific in its measure of the effect of a specific treatment in the form of tumor control or radiographic response. Reviewing the literature and trying to draw conclusions are therefore challenging when both OS and PFS are applied [74]. The response assessment criteria for GBM has been developed over the course of several decades as a result of technology advances in imaging and expanded knowledge on tumor biology. Before 1990, the *Levin and WHO Oncology Response Criteria*, which primarily was based on contrast-enhanced computer tomography, was the standard assessment methods [75]. These were substituted by the standardized *McDonald Criteria*, which took into account that contrast enhancement could be affected by clinical factors such as the use of corticosteroids [76]. The *McDonald Criteria* incorporated the clinical assessment (neurology status) of the patient in the designation of response to therapy as being a complete response, partial response, or stable or progressive disease. With the arrival of magnetic resonance imaging and new therapeutic options, the response assessment criteria was developed further and standardized with regard to all aspects of imaging, timing, and evaluation techniques. Especially the introduction of bevacizumab, a monoclonal antibody targeting VEGF-A and a resulting increased risk of pseudoprogression interpreted as disease progression stimulated the modification and lead to the *Revised Assessment in Neuro-Oncology (RANO) Criteria* in 2010 [77–80]. Because of this development over the last 20 years, caution should be taken in the comparison of particular PFS data.

### Aim of the Review

This review is aimed at providing an up-to-date account of the miRNA expression profiles in tumor tissue associated with prognosis and survival in GBM. It is meant as an updatable list of studies and signatures that have been linked to the progression of GBM, to give an account of the miRNAs, which have been reported to be suitable as a prognostic factor for short- or long-term survival. Plasma miRNA expression has also been associated with survival in GBM, but this is not within the scope of this review [25]. Based on the literature, the studies are stratified into those based on publicly available databases and those conducted on independent tissue cohorts. These data sets have been extensively reviewed and combined to derive a signature or pattern of miRNAs, which has a prognostic potential. The miRNAs reported to have a protective or risk-associated profile have been highlighted in relation to GBM. Finally, the studies that have reported a miRNA signature with respect to prognosis have been compared to find common miRNA profile across the different studies.

## Methodology and Delimitations

A Medline database search on “microRNA, glioblastoma, survival, prognosis and progression” (typed: “(microRNA OR miRNA) AND (glioblastoma OR glioma) AND (survival OR prognosis OR progression)”) was performed (date of last search entry: November 26, 2013). The results contained a total of 270 papers; 125 of these were chosen based on title and abstract content. Of the remaining, 100 papers were cell culture studies and 45 were reviews or review like and were therefore excluded. A total of 125 papers were reviewed for miRNA expression level in GBM correlated to survival and/or progression, 25 involved database studies, and 35 contained studies on GBM tissue (not database-derived). The miRNA profiles, often presented in the form of signatures, were extracted from the papers. This review summarizes the studies investigating miRNAs in GBM and explores their correlation to clinical outcome and highlights the functional characteristics of the miRNAs linked to protection (i.e., longer survival) or risk (i.e., shorter survival). The miRNAs that are included in the signature of more than one study and involved in the progression of GBM have been identified, and their functional role, if known, is discussed.

## Prognostic MicroRNA Signatures in Glioblastoma Multiforme

### MicroRNA Signatures Derived from Database Mining

A total of 25 studies were based on database entries. For the individual studies, the database accessed, cohort size, cohort factor, and normalization methodology along with miRNA signature were documented (Table 1). The majority of the studies used *The Cancer Genome Atlas* (TCGA) (http://cancergenome.nih.gov). However, four studies used the *Chinese Glioma Genome Atlas* (CGGA) (http://www.cgga.org.cn), which uses the *Illumina Human v2.0 miRNA Expression BeadChip* microarray platform [11, 81–83]. The study by Ma et al evaluated two large cohorts of data, the CGGA with 198 samples containing low-grade gliomas and 91 GBMs and an additional cohort of 128 samples, with low-grade gliomas and 68 GBMs to validate the array data. High expression of miR-196b was conferring poor prognosis when stratifying the patients into high miR-196b expression and low miR-196b expression groups [82]. Following a similar experimental setup, Wu et al. looked at 91 GBM patients taken from the array data and validated their findings in a cohort of 60 GBM patients. Here, they focused on miR-328, showing that a low level of expression was conferring poor prognosis [83]. The TCGA dataset has also been used for developing a new method for predicting the outcome based on miRNA expression; however, only one of the studies provided the miRNA identified [84].

**Table 1:** Studies performed on dataset obtained from public databases

Reference	Cohort size	Database	Accessed	Cohort factor	Normalization	Validation	No. of miRNAs studied
Bozdag et al. [89]	385	TCGA	Jul, 2011	Age-specific signature	Level 3		19
Dai et al. [132]	465	TCGA				In vitro, tissue	1
Delfino et al. [71]	253	TCGA	Dec, 2009	miRNA biomarkers of glioblastoma survival	Quantile normalized, collapsed within microRNA, and log2-transformed		45
Gabriely et al. [85]	261	TCGA			Level 2	Tissue, in vitro	1
Genovese et al. [133]	290	TCGA			Level 3	In vitro, xenografting	8
Guessous et al. [86]		TCGA				Tissue, in vitro	1
Haapa-Paananen et al. [134]	308	TCGA			Level 3	Used to validate signature found in cell culture	8
Hua et al. [135]	580	TCGA		Antagonistic activity on cell proliferation and “stemness”			12
Kim et al. [36]	261	TCGA		Classification	Level 3; mean centered, and the STD was normalized to one per array		121
Lee et al. [136]	491	TCGA	Sep, 2011		Level 3	Tissue, in vitro	1
Li et al. [84]	371	TCGA		Method development		Tissue	5
Ma et al. [82]	198	CGGA		Assess prognostic value		Tissue	1
Qiu et al. [137]	480	TCGA			Level 3	Tissue, in vitro	1
Qiu et al. [138]	480	TGCA	Jul, 2013	Signature for GBM survival	Level 3		6
Srinivasan et al. [87]	222	TCGA	Jul, 2010	Signature for GBM survival	Level 1; quantile-normalized and log2-transformed		10
Suzuki et al. [139]	478	TCGA	Mar, 2012	Method development	Level 3; mean centered, and the standard deviation was normalized to one per array	Divided into a training set and a testing set	
Tao et al. [81]	220	CGGA		FOS expression	Illumina BeadStudio Data Analysis software	Tissue, in vitro, xenografting	2
Wang et al. [11]	170	TCGA		IDH1 mutation signature	Level 3		23
Wang et al. [140]	198	CGGA				Tissue, in vitro	1
Wu et al. [83]	198	CGGA		Grade-specific miRNAs		Tissue	1
Xiao et al. [141]	378	TCGA		miRNA-mRNA modules			11
Yin et al. [107]	188	TCGA		EGFR amplification		Tissue, in vitro	1
Zhang et al. [90]	424	TCGA	Feb, 2011	TMZ and MGMT	Level 3	Tissue, in vitro	9
Zhang et al. [91]	345	TCGA		Signature for GBM survival		Tissue	5
Zinn et al. [88]	255	TCGA	Oct, 2011	VAK classification	Level 2, multiarray algorithm	No	8

For each study, the cohort size, the database utilized, the date of accession, and the cohort factor of investigation are stated. The type of normalization used and the levels of data are described; level 1 is the raw data and level 2 or 3 are normalized data from TCGA. Finally, the number of miRNAs investigated is reported

*VAK* Volume, Age, Karnofsky performance score, *MGMT* methyl guanine methyl transferase, *TMZ* temozolomide, *EGFR* epidermal growth factor receptor, *IDH1* isocitrate dehydrogenase 1, *FOS* FBJ murine osteosarcoma viral oncogene homolog, *GBM* glioblastoma multiforme

While a few studies gave rise to a defined multiple-miRNA signature, eight of the studies looked at a single miRNA. For example, two studies evaluated the functionally well-characterized miRNA, miR-10b. Gabriely et al. showed that miR-10b was expressed in GBM tissue while not present in normal brain tissues. Using TCGA data, they investigated the association between the expression of miR-10b and clinical outcome and found that miR-10b correlated with survival although with stratified conditions, the association was insignificant. When the correlation with survival for miR-10b was assessed together with miR-10a, however, the association with survival was significant regardless of stratification; hence, high levels of miR-10 conferred poor survival [85]. Guessous et al. also found a correlation between high levels of miR-10b and poor survival by analyzing the TCGA data and further reported on a functional role of miR-10b in GBM stem cells [86].

Since the majority of the studies use the TCGA dataset in analyzing the expression of miRNAs in GBM, the platform for generating the data was the same. The only differences seen were in the downstream analysis, other clinically prognostic factors, and the type of filtering applied. Expression analysis was conducted on *Agilent 8 × 15 K Human miRNA* microarrays, with data available at four levels. The first level is the raw non-normalized data from the array (level 1), and the second level (level 2) is the processed normalized signal. The third level (level 3) is the segmented data, assemblies of the processed data from single samples, and grouped by probed loci to form larger contiguous regions. The fourth level (level 4) is the summary, a quantified association across classes of samples and associations based on molecular abnormalities, sample characteristics, and/or clinical variability. Not all studies state the level of data they use, but most use the third level. Only one study, looking at a ten-miRNA signature, used level 1 and quantile-normalized the expression data. Here, they segregated patients in to high- and low-risk groups and identified seven miRNAs associated with high risk of disease progression and three miRNAs that were found to be protective [87]. A more elaborate study by Delfino et al. used quantile-normalized data (although it does not specify the level of the data set analyzed) and identified 45 miRNAs in the TCGA data across race, gender, recurrence, and therapy linked to survival [71]. Using level 2 data with multiarray algorithm normalization, Zinn et al. looked at 78 patients and included a *Volume* (tumor volume), *Age*, and *Karnofsky Performance Score* (VAK) classification to dichotomize the patients into VAK A (good prognosis) and VAK B (poor prognosis). A total of five miRNAs were associated with short-term survival (miR-566, miR-505, miR-345, miR-484, and miR-92b), and three miRNAs were associated with long-term survival (miR-511, miR-369-3p, and miR-655) [88].

Though the normalization was not standard for all studies along with the variation in cohort size, most of them used normalized data from the TCGA database and therefore had the same material. The cohort size, however, ranges from 170 to 580 (mean = 329 ± 121) and is a function of the cohort factors that are investigated. For example, factors such as age, grade, MGMT methylation, chemotherapy regimen, IDH1 mutation, or grade sub-classification are just some examples of where the investigation is based on prior knowledge of clinical data correlated with expression and survival [11, 36, 83, 89, 90]. In addition to the TCGA, a number of the studies have used independent GBM tissue validation cohorts for identifying differentially expressed miRNAs with respect to cohort factors [82, 83, 91].

### MicroRNA Signatures Derived from Independent Tissue Cohorts

The studies performed on independent sample sets can generally be characterized as being validation of database findings, validation of literature findings, or novel array-based determination of miRNA profiles of clinical interest in GBM (Table 2). A total of 35 studies identified miRNA signatures associated to survival, and many of these have used tissues to validate signatures previously found in datasets described in the last section and contained in Table 1. The majority of studies (*n* = 30) use PCR-based methods when validating miRNA expression, while several studies use different types of arrays. The PCR-based methods require normalization, and most of the studies use RNU6B, though there are a few studies that use others, such as hsa-miR-16 or RNU5A [51, 92].

**Table 2:** Studies performed on independent tissue cohorts

Reference	Cohort factor	Cohort size	Method	Control	Normalization	Validation	No. of miRNAs studied
Chang et al. [142]		128	RT-qPCR	10 × non-neoplastic brain tissue	RNU6B		1
Chen et al. [143]		43	In situ hybridization RT-qPCR	Normal brain tissue		In vitro	1
Dai et al. [132]		19	RT-qPCR	3 × severe traumatic brain injury		In vitro	1
Gabriely et al. [85]			RT-qPCR	Normal brain tissue		In vitro, xenografting	1
Gao et al. [144]		151	RT-qPCR	15 × severe traumatic brain injury	RNU6B		1
Guan et al. [65]	Grade specific miRNAs	92	PCR array (TaqMan Human miRNA array v1.0 (PE Applied Biosystems)) RT-qPCR	1 × epilepsy 1 × no tumor	RNU44 and RNU48		1
Guessous et al. [86]		20	RT-qPCR	5 × normal brain tissue	RNU6B	In vitro	1
He et al. [145]		112	RT-qPCR	10 × non-neoplstic brain tissue from decompressive craniectomy after brain injury	RNU6B		1
Hermansen et al. [146]		193	In situ hybridization	Not described	RNU6B		1
Hou et al. [147]		102	RT-qPCR	20 × non-neoplastic brain tissue from decompressive craniectomy after suffering brain injury	RNU6B		1
Ilhan-Mutlu et al. [95]	Progression—compare paired primary and secondary	15	RT-qPCR	3 × epilepsy	RNU6B		7
Jiang et al. [148]		253	RT-qPCR, in situ hybridasation	3 × died in traffic accident	RNU6B		1
Jiang et al. [149]		166	RT-qPCR	10 × non-neoplastic brain tissue from decompressive craniectomy after brain injury	RNU6B		1
Lakomy et al. [12]	Methylation and TMZ	38	RT-qPCR	6 × normal AVM and commercial RNA from adult brain			8
Lee et al. [136]			RT-qPCR	Non-neoplastic brain tissue	RNU6B	In vitro	1
Li et al. [84]	Method development	160	Human v2.0 miRNA expression BeadChip	Not described	Log transform		5
Li et al. [150]		128	RT-qPCR	Paired adjacent non-neoplastic brain tissue	RNU6B		1
Lu et al. [151]		108	RT-qPCR	20 × dead from traffic accident	RNU6B		1
Lu et al. [152]		108	RT-qPCR	20 × dead from traffic accident	RNU6B		1
Ma et al. [82]	Assess prognostic value	128	RT-qRCP	Not described			1
Niyazi et al. [94]	Signature for GBM survival	35	Biochip “Geniom Biochip MPEA homo sapiens” (Febit)	Not described			30
Qiu et al. [137]		25	RT-qPCR	14 × non-neoplastic brain tissue	RNU6B	In vitro	1
Quintavalle et al. [92]	MGMT and common classification	34	RT-qPCR	Not described	RNU5A, beta-actin		2
Speranza et al. [153]	NEDD expression		RT-qPCR	Not described	RNU6B	Proliferation and invasion assay, transfection	1
Sun et al. [154]		168	RT-qPCR Taqman miRNA array	21 × cerebral trauma samples	RNU6B		1
Tao et al. [81]	FOS expression	12 50	RT-qPCR In situ hybridasation	3 × normal brain	RNU6B	In vitro, xenografting	2
Wang et al. [112]		108	RT-qPCR	20 × normal controls no pathological lesions	RNU6B		1
Wang et al. [140]		30	RT-qPCR	Severe traumatic brain injury		In vitro, xenografting	1
Wu et al. [83]	Grade-specific miRNAs	100	RT-qPCR	Not described	RNU6B	Validation set	1
Wu et al. [155]		128	RT-qPCR Taqman miRNA array	10 × cerebral trauma samples	RNU6B		1
Yin et al. [107]	EGFR amplification	55	RT-qPCR SNP-chip analysis	Not described	RNU48	Transfection, proliferation, migration, luciferase assay	1
Zhang et al. [96]		50 22	In situ hybridization RT-qPCR	Not described	RNU6B	Transwell assay, wound healing assay, transfection, xenografting	2
Zhang et al. [90]	TMZ and MGMT	82	Illumina Human v2.0 miRNA Expression BeadChip	Not described			9
Zhang et al. [91]	Signature for GBM survival	117	Illumina Human v2.0 miRNA Expression BeadChip	Not described	Average expression	Validation set	5
Zhi et al. [51]	Signature for GBM survival	124	RT-qPCR	60 × normal adjacent tissue	hsa-miR-16	Split into training and validation set	3

For each study, the cohort factor of investigation, the cohort size, the methodology, and the choice of control tissue are stated. The method of normalization, whether it is validated, and the number of miRNAs reported in the study are described

*TMZ* temozolomide, *MGMT* methyl guanine methyl transferase, *EGFR* epidermal growth factor receptor, *RT-qPCR* real-time quantitative polymerase chain reaction, *GBM* glioblastoma multiforme, *AVM* arteriovenous malformation

The starting material used in the studies was either tissue or formalin-fixed paraffin-embedded (FFPE) tissue with a variable cohort size (min = 12, max = 253, mean = 91 ± 59). With regard to sample preparation, de Biase et al. have shown that there is no difference in the miRNA expression obtained from tissue and FFPE tissue, and some studies also use both types to validate their findings [93]. Twenty-five studies focused on single-miRNA candidates, while the remainder focused on expression profiles of several miRNAs (min = 1, max = 30, mean = 3 ± 5).

While most of the studies focus on smaller miRNAs signatures, Niyazi et al. present a larger cohort of miRNA as a putative survival signature. They used a top-down approach, where they filtered the miRNAs based on the variance in expression across the samples and chose the 30 most dysregulated miRNAs. These miRNAs were used to stratify the samples into two patterns, which correlated with short- and long-term survival [94]. This approach was also applied in several database studies, limiting the number of miRNAs down to a specific signature [51, 84, 90, 91]. Others looked at pre-selected miRNAs already linked to GBM pathogenesis in the literature [92, 95, 96]. Zhang et al. found that miR-221 and miR-222 expression was significantly increased in high-grade gliomas compared with low grade, positively correlated with degree of glioma infiltration. This corresponded well to the fact that overexpression of miR-221 and miR-222 increased cell invasion [12]. In addition, Quintavalle et al. showed that miR-221 and miR-222 were upregulated in GBM patients and that they target MGMT mRNA thereby inducing greater temozolomide-mediated cell death [92].

Of all the studies, 12 of them utilized both databases and independent tissue cohorts. They all link an expression of one or more miRNAs to survival; however, some studies categorize a given miRNA to be protective or risk-associated. Three studies used hazard ratio to assess whether a specific miRNA was protective or risk-associated, while Wang et al. used a *Significance analysis of microarray* (SAM) and Li et al. used the Cox-regression coefficient to designate the miRNAs [11, 84, 87, 90]. Interestingly, large variations can be found in the choice of control tissues across the individual studies, ranging from purchased RNA from normal brains to tissues from epilepsy patients or patients with cerebral trauma. Such differences in control tissues might also be a factor in the incoherency between the miRNA signatures found in the different studies (Table 2). In addition, only few studies specify their use of the terms OS and PFS, which also makes direct comparison difficult.

## MicroRNA Reported to be Protective or Risk-Associated

A number of studies provided miRNA signatures associated with survival or progression in GBM and reported that individual miRNAs of these signatures could be regarded as either protective or risk-associated. These miRNAs and their functional role in GBM pathogenesis and progression are presented in Table 3.

**Table 3:** miRNAs reported to be protective or risk-associated

miRNA	Reference	Function in GBM	Validated targets	Reference
*hsa-miR-9*	[112]	Overexpressed (5), oncogenic properties	CAMTA1	[39, 40, 64, 66, 156]
*hsa-miR-17-5p*	[87, 112]	Overexpressed (9), oncogenic properties	POLD2, TGFβ-RII, CTGF, CAMTA1, PTEN	[39, 40, 42, 66, 67, 97, 156, 157]
*hsa-miR-19a*	[112]	Disputed expression in GBM (6)	CTGF	[39, 42, 66, 67, 97, 157]
*hsa-miR-19b*	[112]	Disputed expression in GBM (4)		[42, 66, 67, 157]
*hsa-miR-20a*	[87, 112]	Overexpressed (5), oncogenic properties	TGFβ-RII, CTGF	[39, 40, 42, 67, 97]
*hsa-miR-99a*	[112]	Overexpressed (2), oncogenic properties		[66, 67]
*hsa-miR-106a*	[87, 112]	Disputed expression in GBM (5)	E2F1	[42, 48, 51, 66, 98]
*hsa-miR-128a*	[112]	Underexpressed (13), tumor suppressive	WEE1, p70S6K1, Msi1, E2F3a, Bmi-1, EGFR, PDGFRα	[12, 40–42, 48, 56, 62, 66, 67, 99–103]
*hsa-miR-128b*	[112]	Underexpressed (7)	WEE1	[41, 42, 48, 56, 62, 67, 158]
*hsa-miR-139*	[112, 159]	Underexpressed (5)		[40, 42, 60, 67, 158]
*hsa-miR-181d*	[90, 160]	Underexpressed (1), tumor suppressive	Bcl-2, K-Ras	[104, 105]
*hsa-miR-183*	[112]	Underexpressed (2)		[42, 64, 67, 148, 161]
*hsa-miR-217*	[112]	Overexpressed (2)		[42, 67]
*hsa-miR-301*	[112]	Overexpressed (2)		[42, 67]
*hsa-miR-324-5p*	[112]	Overexpressed (1)		[56]
*hsa-miR-328*	[83]	Underexpressed (2)		[39, 42]
*hsa-miR-374*	[112]	Overexpressed (1)		[40, 66]
*hsa-miR-497*	[112]	Overexpressed (1)		[67]
*hsa-miR-524-5p*	[84, 90]	Overexpressed (1)		[67]
*hsa-miR-544*	[84]	Overexpressed (1)		[67]
*hsa-miR-628-5p*	[84]			[66]
*hsa-miR-1227*	[90]	No studies		
**hsa-miR-15a**	[159]	Overexpressed (4)		[39, 62, 66, 67]
**hsa-miR-31**	[87]	No studies		
**hsa-miR-34a**	[112]	Underexpressed (5), tumor suppressive	SIRT1, c-Met, Notch1/2, PDGFRA, Msi1	[100, 106, 162–164]
**hsa-miR-34b**	[112]	No studies		
**hsa-miR-146b**	[87]	Underexpressed (5), tumor suppressive		[108, 109]
**hsa-miR-148a**	[87, 112]	Overexpressed (1)		[64]
**hsa-miR-155**	[112]	Overexpressed (6)		[40, 42, 45, 66, 67, 165]
**hsa-miR-193a**	[87]	Overexpressed (1)		[42, 67]
**hsa-miR-200b**	[87]	Overexpressed (2)		[67]
**hsa-miR-221**	[87, 112]	Overexpressed (11), oncogenic properties	P27, Akt, PUMA, P57, PTPμ, Cx43, TIMP3, MGMT	[41, 48, 62, 63, 110, 111, 113, 166–169]
**hsa-miR-222**	[87, 112]	Overexpressed (9), oncogenic properties	P27, Akt, PUMA, P57, PTPμ, Cx43, TIMP3, MGMT	[41, 48, 62, 110, 111, 166–169]
**hsa-miR-297**	[91]	No studies		
**hsa-miR-299-3p**	[91]	Underexpressed (1)		[42]
**hsa-miR-346**	[91]	No studies		
**hsa-miR-518b**	[91]	Overexpressed (1)		[67]
hsa-miR-541*	[91]	No studies		
**hsa-miR-551a**	[91]	No studies		
**hsa-miR-566**	[91]	Overexpressed (1)		[67]
**hsa-miR-661**	[91]	Overexpressed (1)		[67]
**hsa-miR-768-3p**	[112]	Overexpressed (1)		[67]
**hsa-miR-936**	[91]	No studies		
**hsa-miR-1238**	[91]	No studies		

MicroRNAs described as either protective (ital) or risk-associated (bold) compared with their corresponding functional characteristics. The terms overexpressed and underexpressed refers to miRNA expression data comparing GBM samples to normal brain tissue. Disputed expression signifies that different studies present contradictory results. The numbered parentheses are numbers of studies supporting the observation

### Clinically Protective MicroRNAs

In the group of the protective miRNAs (*n* = 22), only two miRNAs, miR-544 and miR-1227, have not been described previously in relation to miRNA alterations in GBM pathogenesis. Eleven of the protective miRNAs were significantly increased in studies comparing GBM specimens to normal brain tissue, while only six were significantly downregulated. Surprisingly, three of these miRNAs are well described as miRNAs with an oncogenic potential and have several validated targets considered to be tumor suppressor genes. This includes the extensively investigated miR-17-5p, which in vitro has been shown to increase angiogenesis and growth when overexpressed and decrease viability and proliferation when inhibited, making it unlikely that this miRNA, at least solely, should be considered protective [97]. As to miR-19a, miR-19b, and miR-106a, there is currently an inconsistency in the literature regarding their role in GBM development. The functional data available on miR-106a shows that overexpression by transfection of GBM cell lines causes a significant decrease in proliferation and an increase in apoptosis, likely mediated by the suppression of E2F1, supporting the notion of it being tumor suppressive [98]. More in line with what would be expected, miRNAs with previously investigated tumor suppressive capabilities are present on the list whereby miR-128a and miR-181d are most notable. miR-128 has been investigated in 13 studies demonstrating its wide range of oncogenic mRNA targets and its ability to inhibit angiogenesis and proliferation and even to significantly decrease total tumor volume in vivo [12, 40–42, 48, 56, 62, 66, 67, 99–103]. Similarly, although, less extensively investigated is miR-181d, which has been shown to target the oncogenes Bcl-2 and K-Ras whereby apoptosis is increased and proliferation decreased. miR-181d transfection is demonstrated to decrease in vivo tumor size and has been shown to increase the susceptibility to the chemotherapeutic agent, temozolomide [104, 105].

### Risk-Associated MicroRNAs

Within the cohort of miRNAs described as risk-associated (*n* = 22), nine have not been previously associated with miRNA modulation in GBM. Of the 13 miRNAs mentioned in the literature, ten are overexpressed in GBM specimen, three are underexpressed, and four have been functionally characterized. miR-34a is well studied in numerous GBM cell lines and shown to increase cell differentiation and decrease total tumor volume in a xenograft mouse model of GBM [106, 107]. The less investigated miR-146b is similarly known to decrease in vitro invasiveness, migration, proliferation, and tumor volume in mice [108, 109]. Both miR-34a and miR-146b are, in terms of isolated functional characteristics, not associated with risk of GBM progression (Table 3). The oncogenic miRNAs, miR-221 and miR-222, clinically associated with risk, have been studied in relation to a diverse list of cancers including GBM. They inhibit a number of common gene targets such as PUMA and P57 both involved in apoptosis. When overexpressed in vitro, both miR-221 and miR-222 potentiate classic cancer hallmarks, i.e., proliferation, angiogenesis, and invasion. In vivo studies have revealed that miR-221 or miR-222 overexpression is associated with increased tumor growth, a situation that can be reversed with administration of corresponding antagomirs [110, 111].

Out of 44 miRNAs reported to be protective or risk-associated, only eight were not previously described as significantly modulated in GBM samples. This demonstrates a relatively broad coverage in terms of the miRNAs investigated purely to assess miRNA modulation in GBM pathogenesis without correlating the data to clinical outcome (Table 3). No general patterns apply to these cohorts of protective and risk-associated miRNAs, as such, several miRNAs, which are described as oncogenic from a functional standpoint, are present within the cohort of protective miRNAs and vice versa. This comparison between in vitro functionality and clinical implication of GBM-related miRNAs illustrates that although a specific miRNA may have a specific set of functional characteristics when artificially over or underexpressed in isolated in vitro models, this is not necessarily a good indicator for the multifactorial clinical progression of GBM. For more elaborate details of the functional characterization of miRNAs involved in GBM, please refer to Møller et al. [38].

## MicroRNAs Included in Several Signatures

Based on the multiple-miRNA signatures identified from both tissue and database studies pertaining to survival, the miRNAs found in multiple studies were identified (Table 4). Most of the miRNA found in signatures are specific for the given study. Thirteen of the miRNAs were identified in more than one study; however, no miRNAs were identified in more than three studies. Through miRNA array analysis, Niyazi et al. found a 30-miRNA signature in an independent cohort, which divided the samples into short- and long-term survival [94]. Furthermore, Zhang et al. and Srinivasan et al. used similar methods and the same database, but the overlap between these studies was poor [87, 91].

**Table 4:** miRNA signatures correlating with survival in GBM

Reference	miRNA
Bozdag et al. [89]	Ebv-miR-BART1-5p, Ebv-miR-BHRF1-2, Hcmv-miR-UL70-5p, hsa-miR-142-3p, hsa-miR-142-5p, hsa-miR-147, hsa-miR-223, hsa-miR-302c, hsa-miR-325, hsa-miR-422b, hsa-miR-453, hsa-miR-507, hsa-miR-552, hsa-miR-558, hsa-miR-620, hsa-miR-649, *hsa-miR-661*
Hua et al. [135]	*hsa-miR-19a*, hsa-miR-93, *hsa-miR-221*, *hsa-miR-222*
Lakomy et al. [12]	*hsa-miR-21*, *hsa-miR-128a*, hsa-miR-181c, *hsa-miR-195*, hsa-miR-196a, hsa-miR-196b, hsa-miR-221, *hsa-miR-222*
Li et al. [84]	hsa-miR-15a, hsa-miR-139-5p, *hsa-miR-524-5p*, hsa-miR-544, hsa-miR-628-5p
Niyazi et al. [94]	hsa-let-7a, hsa-let-7f, hsa-let-7g, hsa-let-7i, hsa-miR-26a*, hsa-miR-29b, hsa-miR-30b, hsa-miR-124, hsa-miR-129-3p, hsa-miR-136, *hsa-miR-195*, *hsa-miR-210*, hsa-miR-374b, hsa-miR-409-3p, hsa-miR-487b, hsa-miR-539, hsa-miR-555, hsa-miR-578, hsa-miR-590-3p, hsa-miR-595, hsa-miR-720, hsa-miR-1260, hsa-miR-1282, hsa-miR-1286, hsa-miR-1305, hsa-miR-2113, hsa-miR-3065-3p, hsa-miR-3132, hsa-miR-3163, hsa-miR-4286
Qiu et al. [138]	hsa-miR-130a, *hsa-miR-155*, *hsa-miR-210*, hsa-miR-323, hsa-miR-326, hsa-miR-329
Srinivasan et al. [87]	*hsa-miR-17-5p*, *hsa-miR-20a*, hsa-miR-31, *hsa-miR-106a*, hsa-miR-146b, *hsa-miR-148a*, hsa-miR-193a, hsa-miR-200b, *hsa-miR-221*, *hsa-miR-222*
Wang et al. [11]	hsa-miR-9, *hsa-miR-17-5p*, *hsa-miR-19a*, hsa-miR-19b, *hsa-miR-20a*, hsa-miR-34a, hsa-miR-34b, hsa-miR-99a, hsa-miR-106a, *hsa-miR-128a*, hsa-miR-128b, hsa-miR-139, *hsa-miR-148a*, *hsa-miR-155*, hsa-miR-183, hsa-miR-217, *hsa-miR-221*, *hsa-miR-222*, hsa-miR-301, hsa-miR-324-5p, hsa-miR-374, hsa-miR-497, hsa-miR-768-3p
Zhang et al. [90]	*hsa-miR-181d*, hsa-miR-297, hsa-miR-299-3p, hsa-miR-346, hsa-miR-541*, hsa-miR-551a, *hsa-miR-661*, hsa-miR-936, hsa-miR-1238
Zhang et al. [91]	*hsa-miR-181d*, *hsa-miR-566*, *hsa-miR-524-5p*, hsa-miR-518b, hsa-miR-1227
Zhi et al. [51]	*hsa-miR-21*, *hsa-miR-106a*, hsa-miR-181b
Zinn et al. [88]	hsa-miR-92b, hsa-miR-345, hsa-miR-369-3p, hsa-miR-484, hsa-miR-505, hsa-miR-511, *hsa-miR-566*, hsa-miR-655

Overview of the miRNA signatures reported in database studies and independent tissue cohort studies correlated with survival or progression of GBM. The miRNAs marked in ital were detected in two or more studies

It is striking that the database studies do not reveal better coherency; however, this could be attributed to the cohort factors studied or the filtering of the miRNAs during the analysis. The tissue studies represent independent cohorts; however, many of the database studies have validated their results in independent cohorts, whereby the cohort factors could be the prime source for the lack of overlap. Additionally, Ilhan-Mutlu et al. chose to investigate seven well-characterized miRNAs (miR-10b, miR-21, miR-181b, miR-181c, miR-195, miR-221, miR-222) and found that none of them correlated with survival [95] contradicting other studies [87, 112]. Therefore, the 13 miRNAs identified in more than one signature could be more applicable in their prediction of survival and of great interest in relation to GBM prognosis.

### Functional Analysis of the MicroRNAs Included in Several Signatures

The majority of the 13 miRNAs included in more than one signature have been functionally characterized in GBM and associated with the expression of validated target genes (Table 5). The most well-characterized miRNA in GBM is miR-21, which functions as an oncogenic miRNA. miR-21 has numerous validated target genes that it represses in GBM and therefore it is interesting that this miRNA is included in two signatures. The target genes of miR-21 include genes associated with proliferation (e.g., PTEN and PDCD4), invasiveness (e.g. TIMP3 and RECK), and susceptibility to chemo and radiation therapy (e.g., hMSH2), factors, which are all characteristics of GBM tumors [44, 48, 59, 70]. The same characteristics of GBM tumor growth are also modulated by miR-221 and miR-222, both of which appear in three signatures. Being less well characterized than miR-21, miR-221 and miR-222 still have several validated target genes including some important tumor suppressor genes such as P27, P57, TIMP3, and Cx43 [96, 111, 113, 114].

**Table 5:** Functional characteristics of miRNAs found in several signatures

microRNA	Validated targets	Functional role when 1: overexpressed, 2: inhibited	No. of signatures included	Reference to functional studies
hsa-miR-106a	E2F1, SLC2A3	1: proliferation↓, apoptosis↑	2	[98, 132]
hsa-miR-136	AEG-1, Bcl-2	1: apoptosis↑	2	[117]
hsa-miR-148a	No validated targets	No functional analysis performed	2	
hsa-miR-155	GABRA-1, FOXO3a	1: proliferation↑, apoptosis↓, invasion↑	3	[170, 171]
hsa-miR-17-5p	POLD2, TGFβ-RII, CTGF, CAMTA1, PTEN	1: angiogenesis↑, growth↑, invasion↑, migration↑, chemosensitivity↓ 2: viability↓, apoptosis↑, proliferation↓	2	[39, 97, 156, 157, 172]
hsa-miR-181b	FOS, MEK1, IGF-1R	1: xenograft growth↓, chemosensitivity↑, invasion↓, proliferation↓, migration↓	2	[81, 173, 174]
hsa-miR-195	E2F3, CCND3, Cyclin D1, Cyclin E1	1: invasion↓, proliferation↓, xenograft growth↓	3	[115, 116]
hsa-miR-20a	TGFβ-RII, CTGF	1: angiogenesis↑, growth↑ 2: viability↓, proliferation↓	2	[97, 157]
hsa-miR-21	RECK, TIMP3, APAF1, ANP32A, SMARCA4, Caspases, PTEN, Cdc25A, HNRPK, TAp63, Spry2, LRRFIP1, PDCD4, hMSH2	1: invasiveness↑, radiosensitivity↓ 2: invasiveness↓, apoptosis↑, viability↓, proliferation↓, in vivo tumor volume↓, chemosensitivity↑, radiosensitivity↑	2	[41, 43, 44, 46–50, 52–55, 58, 59, 68, 70, 175]
hsa-miR-210	No validated targets	No functional analysis performed	2	
hsa-miR-221	P27, Akt, PUMA, P57, PTPμ, Cx43, TIMP3, MGMT	1: proliferation↑, invasiveness↑, in vivo tumor volume↑, apoptosis↓, migration↑ 2: proliferation↓, apoptosis↑, in vivo tumor volume↓, radiosensitivity↑	3	[92, 96, 110, 111, 113, 114, 167, 168, 176]
hsa-miR-222	P27, Akt, PUMA, P57, PTPμ, Cx43, TIMP3, MGMT	1: proliferation↑, invasiveness↑, in vivo tumor volume↑, apoptosis↓, migration↑ 2: proliferation↓, apoptosis↑, in vivo tumor volume↓, radiosensitivity↑	3	[92, 96, 110, 111, 113, 114, 167, 168, 176]
hsa-miR-566	No validated targets	No functional analysis performed	2	

miRNAs found in several signatures and their functional characteristics. Each miRNA is noted along with their validated targets, their functional role, and how many signatures they appear in. Regarding the functional role, 1 designate the functional role of the miRNA when it is overexpressed and 2 the functional role when it is inhibited in vitro or in vivo

In addition to the oncogenic miRNAs identified in more than one signature, different tumor suppressor miRNAs were also found in several signatures. miR-195 has validated target genes, including some cyclins and E2F3, which are associated with cell proliferation [115, 116]. Hence, a low expression of this miRNA should in theory correlate with a favorable clinical outcome, which is in fact reflected in the clinical data [12]. Another interesting miRNA shown in more than one signature is miR-136, which has very little functional characterization, but the current validated target genes include the important oncogene, Bcl-2 [117]. Several of the 13 miRNAs (miR-155, miR-17-5p, miR-181b, miR-195, miR-20a, miR-21, miR-221, and miR-222) are known to modulate the mesenchymal mode of migration and invasion (MMMI), which is an important characteristic of GBM cells [38, 118]. Three of the miRNAs identified in more than one signature have no functional characterization and could possibly reveal numerous relevant target genes to substantiate the importance of the 13 miRNAs in future determination of patient prognosis.

## Perspectives on MicroRNA-Based Therapies for the Treatment of Glioblastoma Multiforme

Given the fact that several miRNA signatures associated with OS or PFS have been identified and that these miRNAs have functional characteristics with importance in GBM progression, a therapeutic concept taking advantage of such correlations seems inherent. The use of miRNA-based therapies in the treatment of GBM is still in its primary phases with exciting basic research being published frequently [119].

Approaches for utilizing miRNAs in such treatment regimens includes both inhibition of oncogenic miRNAs (e.g., miR-21) or overexpression of tumor suppressor miRNAs (e.g., miR-146b) with different types of carriers to facilitate delivery directly to the tumor tissue [120, 121]. Systemic administration of a liposome-encapsulated tumor suppressor miRNA, miR-7, led to a significant tumor size reduction in a xenograft mouse model of GBM. In addition, several key oncogenes were downregulated upon the tumor suppressor miRNA delivery [122]. Another more sophisticated type of lipid-based delivery was exploited by Griveau et al. where locked nucleic acid miRNA inhibitors against miR-21 conferred increased radiosensitivity in U87MG cells [120]. miRNA carriers have also been generated with polymer-based technology, using poly(amido amine) to encapsulate miR-7 for delivery to U251 cells, which resulted in a higher transfection efficiency than liposomal delivery [123].

Of particular interest in solving the problems with efficient drug delivery to the brain, both in malignancies and neurodegenerative diseases are the use of exosomes as drug carriers [124]. Exosomes are endogenous vesicular structures with a diameter ranging from 40 to 120 nm produced by all cells in the body [125]. They are characterized by expression of specific proteins in the membrane (especially tetraspanins) and their ability to deliver proteins, mRNA and miRNAs [126]. The delivered mRNAs and miRNAs are fully functional and can be translated into protein or inhibit mRNA targets in the recipient cells [127, 128].

The potential of exosomes to deliver functional RNAs to cells was utilized by Alvarez-Erviti et al., who provided interesting evidence as to how exosomes might be used to deliver drugs across the ever troubling blood-brain barrier. Immature dendritic cells were transfected to produce exosomes that expressed a neuron-specific targeting peptide on their surfaces to facilitate specific delivery of the exosome cargo. These exosomes successfully delivered both GAPDH- and BACE1-siRNA across the blood-brain barrier resulting in specific gene silencing in the neuronal tissue [129]. Using a somewhat similar approach, Ohno et al. showed that exosomes targeted to EGFR could deliver the tumor suppressor miRNA, let-7a, to a xenograft breast cancer model after intravenous administration. Furthermore, let-7a suppressed the growth of the tumor underscoring the relevance of using exosomal delivery in malignant diseases [130].

Evidence is now emerging showing that exosomal delivery of interfering RNAs could be relevant in the treatment of GBM. GBM cell lines were shown to be resistant to treatment with anti-miRs against the oncogenic miRNA, miR-9, described in Table 3. However, if these GBM cells were co-cultured with anti-miR-transfected mesenchymal stem cells (MSCs) or cultured in the presence of anti-miR-transfected MSC-exosomes, miR-9 was significantly downregulated. This decrease in miR-9 expression made the GBM cells more susceptible to treatment with the chemotherapeutic drug, temozolomide [131]. Katakowski et al. also produced exosomes in MSCs, which were transfected with a miR-146b expression vector. The resulting miR-146b-containing exosomes were injected into xenograft GBM tumors, leading to a significant reduction in tumor volume compared to vehicle-treated controls [121]. Interestingly, it has previously been shown that miR-146b negatively correlates with survival in GBM [87]. The use of exosomes in the treatment of GBM may have a great potential and should be substantiated with more evidence including choice of relevant miRNA cargo and direct targeting of GBM cells to facilitate intravenous administration.

## Concluding Remarks

This review presents the studies investigating the expression of specific miRNAs or miRNA signatures with respect to their correlation to clinical progression of GBM. A large part of the studies utilize data from the same databases (TCGA or CGGA), but they do not necessarily reveal the same results. This is because the extracted data and the filtering based on clinical information differ across individual studies, which makes comparison difficult (Table 1). The studies using individual tissue cohorts also reveal different miRNA signatures with only some consistency between them. Such varying results may be caused by several factors, including miRNAs investigated, type of array platform utilized, cohort size, and especially the choice of control tissue. Comparing miRNA expression data to control tissue obtained from another type of diseased brain (i.e., epilepsy) might be problematic because it may induce variations in the miRNA expression data compared to studies using non-diseased normal brain tissue. Furthermore, imperfect description of terminology with regard to OS and PFS may also add complexity to the comparison of the different miRNA signatures. Several studies report some miRNAs to have a protective or risk-associated profile with respect to their correlation with clinical outcome in GBM. Interestingly, several of these miRNAs have validated functions in vitro and in vivo, which are opposite to the way that they should mediate either protection or risk. Therefore, the in vitro and in vivo studies available for numerous miRNAs are not necessarily good indicators for the multifactorial clinical progression of GBM (Table 3). However, many of the miRNAs reported to be either protective or risk-associated or the miRNAs included in several signatures do in fact have validated targets and functional characteristics, which are in line with their correlation to clinical progression or survival of GBM (Tables 3, 4, and 5). Having been associated with disease progression or survival in several studies, these miRNAs may be valuable for future determination of patient prognosis and could possibly serve as targets for miRNA-based therapies, which hold a great potential in the treatment of this severe malignant disease.
